# A systematic review of sample representativeness and homogeneity in exercise trials using group designs for people with cerebral palsy

**DOI:** 10.1016/j.jsampl.2025.100103

**Published:** 2025-05-20

**Authors:** S.M. Tweedy, I.M. Dutia, L. Caughey, B. Demetriou, E.M. Beckman, J. Cairney

**Affiliations:** a University of Queensland, School of Human Movement and Nutrition Sciences, Australia; b Australian Catholic University, School of Allied Health, Australia

**Keywords:** Cerebral palsy, Exercise, Representativeness, Homogeneity

## Abstract

**Background:**

Evidence quality from exercise trials which use group designs is partly dependent on whether study samples represent the population and acceptable sample homogeneity is achieved. This study aimed to review studies evaluating exercise training for people with cerebral palsy (CP) and appraise to what extent i) participants were representative of people with CP; and ii) internal validity was threatened by sample heterogeneity.

**Methods:**

A search of 5 major databases was conducted to identify exercise trials which use group designs for people with CP. Participant characteristics were extracted and used to divide participants into sub-populations. Indicative Participant Prevalence Ratio (iPPR) was calculated to assess representativeness. Sample homogeneity was appraised for each study.

**Results:**

Fifty-one studies evaluating 836 participants were appraised. Adults comprise 60 ​% of the CP population but were grossly underrepresented (iPPR<0.4). Older adults, people with dyskinetic and ataxic CP and wheelchair users were also grossly underrepresented. The number of studies that accounted for key prognostic variables was: age - 26 studies (51 ​%); sex - 0 studies (0 ​%); neurological subtype - 21 studies (41 ​%); functional effects - 14 studies (27 ​%) and comorbidities - 1 study (2 ​%).

**Conclusion:**

Adults with CP and people with high support needs are underrepresented, and future research should prioritise this subpopulation. Trials using group designs require researchers to reconcile two competing interests – adequate sample size and sample homogeneity and to date, sample homogeneity has received insufficient priority. This threatens internal validity and, consequently, the overall quality of evidence underpinning clinical exercise prescription is likely to be lower than previously thought.

**Key points**

What is already known?

• The exercise training responses of people with cerebral palsy (CP) are a function of the interaction between key prognostic variables including their age, sex, neurological subtype, functional effects and type and severity of comorbidities.
• Evidence quality from exercise training studies using group designs will depend on the extent to which study samples represent the CP population; and studies achieve acceptable sample homogeneity.

What are the new findings?

• Children and adolescents are over-represented in the literature, and adults, wheelchair users, people with dyskinetic or ataxic CP and certain comorbidities are grossly under-represented.
• Sample heterogeneity limits the internal validity of most exercise training studies of people with CP identified in this review, including RCTs.
• For clinical practice, this means that the overall quality of evidence is likely lower than previously thought. Exercise recommendations for people with CP based on existing evidence should be interpreted cautiously, particularly in under-represented subpopulations.

## 1 Background

Exercise training improves health-related fitness (e.g., cardiorespiratory fitness and muscular strength) in the general population [1]. Age and sex are both recognised prognostic variables in exercise trials – ‘characteristics which can predict a participant's response to an intervention’ [2]. For example, a resistance training program will elicit different responses in prepubescent female children, compared with adult males [3]. In studies using group designs, which organise participants into one or more distinct groups, manipulate conditions and report outcomes at a group level, internal validity will be threatened if the included participants are heterogeneous in relation to age and sex. Importantly, the threat is independent of the randomisation of participants to groups [4].

Cerebral palsy (CP) is the most common childhood neurological disorder, caused by a non-progressive injury to the developing foetal or infant brain [5]. CP affects exercise training responses – defined as the physiological and functional changes that occur in response to structured and repeated physical training (for example, cardiovascular fitness, muscle strength, muscle power or endurance). The volume of research investigating these effects in individuals with CP is increasing [[6], [7], [8], [9]].

However, CP is not a single or unitary diagnostic category. CP is defined as “an umbrella term encompassing a heterogeneous group of permanent but not unchanging disorders of movement and posture” [10]. For example, people with CP can be classified based on one of seven neurological subtypes, each of which are recognised in the ICD 10. These are: spastic quadriplegic (16.3 ​%), spastic diplegic (28.8 ​%), spastic hemiplegic (35 ​%), dyskinetic, incorporating dystonia and choreoathetotic [11] (5.6 ​%), ataxic (4.0 ​%), mixed (1.3 ​%) and unspecified (9 ​%) [12]. Each subtype varies in terms of severity and motor distribution (i.e. parts of the body affected).

CP is also characterised by heterogenous functional effects. There are classification systems for at least six different types of function, these being the Gross Motor Function Classification System (GMFCS), the Manual Ability Classification System (MACS), the Eating and Drinking Ability Classification System (EDACS) [13], the Bimanual Fine Motor Function Classification System (BFMF) [14], the Visual Function Classification System (VFCS) and the Communication Function Classification System (CFCS) [15]. Each system has five levels ranging from I (least severe) to V (most severe). Full descriptions of each of these classification systems are reported elsewhere [16].

In addition to the heterogeneity associated with varying combinations of neurological subtype, motor distribution and functional effects, 95 ​% of people with CP have at least one co-morbidity [12].Co-morbidities are defined in this context as a standalone disorders that are often associated with CP but which can also occur in people without CP. Some of the most common are known or potential prognostic variables, including intellectual disability (50 ​%), epilepsy (39.0 ​%), digestive system diseases (39.1 ​%), malnutrition and eating difficulties (23.0 ​%), scoliosis (15 ​%) and pain (75 ​%) [12].

We posit that the clinical characteristics of CP described above – neurological subtype, level of functioning in different domains and the range of comorbidities – constitute three distinct groups of prognostic variables. It follows that the exercise training responses of any individual with CP will be a function of interaction between the constellation of CP-specific prognostic variables they are affected by as well as their age and sex.

Based on this position, in order to draw conclusions about responses to active exercise training in people with CP, study samples should be representative of the diversity of clinical presentations in the CP population. To the extent that samples are not representative, knowledge will be incomplete. Additionally, studies using group designs require samples that are homogeneous in relation to these defining prognostic variables. To the extent that such homogeneity is not achieved, the internal validity of the study will be threatened by predictable systematic between-participant differences in exercise training responses [17].

Accordingly, the aim of this study was to systematically identify studies that have used group designs to evaluate exercise training responses in people with CP and appraise.

1) the extent to which the people with CP who have been included in exercise training studies are representative of the CP population; and
2) whether, and to what extent, the internal validity of each study was threatened by participant heterogeneity in relation to age and sex, as well as in the three key groups of clinical prognostic variables affecting people with CP – neurological subtype, functional effects and comorbidities.

## 2 Methods

This study followed PRISMA systematic review guidelines and was registered on the Open Science Framework (OSF) (register number/DOI: 10.17605/OSF.IO/64U5B).

### 2.1 Search strategy

An initial limited search was conducted on PubMed and EMBASE to identify relevant key words (e.g., “cerebral palsy”) and truncations (e.g., exercis∗). Five electronic databases were then searched (PubMed, EMBASE, Medline CINAHL, Web of Science and PEDro) in March 2022 and again in July 2024 (See Appendix 1). Search filters were used to ensure articles retrieved met the inclusion and exclusion criteria.

### 2.2 Source of evidence screening and selection

Articles were exported and duplicates removed. Two reviewers (from LC, ID & BD) screened title and abstracts and, for those included, then conducted a full text review. Conflicts were resolved via discussion between two reviewers or discussion with a third reviewer when required.

Studies meeting following criteria were included: (1) participants with a diagnosis of CP; (2) the design comprised one or more groups receiving the intervention; (3) exercise training based primarily on voluntary movement was performed for >6 weeks; (4) the training specifically measured and aimed to improve health-related fitness (i.e., cardiorespiratory fitness, muscular strength, muscle power or endurance); (5) original investigation published from January 1980 in peer-reviewed literature in English. Studies were excluded if: (1) the exercise training included any form of assistance, active or passive (e.g., motorised ergometer or functional electrical stimulation); (2) results from participants with CP were not reported separately; (3) observational designs or qualitative methods were used; or (4) the full article was unavailable. A backwards search of reference lists from included studies was conducted to identify any studies missed.

### 2.3 Data extraction and appraisal

The following data was extracted: first author, year and title, total number of participants in the intervention group, participant age, sex, neurological subtype, functional effects, and comorbidities. Each study's inclusion/exclusion criteria were extracted and reported in three categories: relevant to intervention safety/fidelity (e.g., intellectual disability or contraindications to exercise), competing interventions (e.g., surgery or botulinum toxin), and baseline physical activity levels (level of activity prior to intervention). These were not participant characteristics but specified which participants were eligible, thereby contributing to the internal validity of the participant group/s.

For each study, five groups of participant characteristics were extracted and reported as follows.

a) Age, categorised as middle childhood (aged 6–12 years), adolescence (age 13–17 years), adulthood (aged 18–64 years) or older adulthood (>64);
b) Sex, categorised as male or female;
c) Neurological subtype categorised as hypertonia (including quadriplegia, diplegia and hemiplegia), dyskinetic (including dystonia and choreoathletosis), ataxic or mixed;
d) Functional effects, categorised on a I to V scale using one or more of the following classification systems [16]: GMFCS; MACS; BFMFCS; EDACS; VFCS; CFCS; or other; and
e) Co-morbidities.

The internal validity of an exercise training study using a group design will be enhanced when participants are homogeneous in relation to the categories of age, sex, neurological subtype, functional effects and co-morbidities. Internal validity will be threatened when participants are heterogeneous in relation to the five key groups of participant characteristics. The term ‘Not Reported’ was used to indicate that a study had not reported a category of participant characteristic and that the participants could therefore be heterogeneous regarding that characteristic.

For those participant subcategories with published estimates of prevalence in the CP population, indicative participant to prevalence ratio (iPPR) was also calculated using the following formula, as previously described [18,19]:

$$\text{iPPR}=\frac{\text{Percentage}\;\text{of}\;\left[\text{CP}\;\text{Population}\;\text{sub}-\text{category}\;\text{name}\right]\;\text{in}\;\text{exercise}\;\text{trials}\;\text{using}\;\text{group}\;\text{designs}}{\text{Estimated}\;\text{percentage}\;\text{of}\;\left[\text{CP}\;\text{Population}\;\text{sub}-\text{category}\;\text{name}\right]\;\text{in}\;\text{the}\;\text{CP}\;\text{population}}$$

An iPPR of ≤0.8 indicated people with CP in the named subcategory were underrepresented in the exercise training literature and 0.4 indicated gross under-representation. An iPPR of >1.2 indicated overrepresentation and 2.0 indicated gross over-representation.

Appraisal of sample homogeneity entailed two stages. The first stage was determining which of the prognostic variables had been reported by the authors. The second stage was determining which of the prognostic variables had been accounted for through trial conduct either explicitly through statistical adjustment or inclusion/exclusion criteria; or implicitly through the characteristics of the participants, in order to achieve sample homogeneity. This critical appraisal approach, which differentiates limitations in reporting from limitations in trial conduct, is consistent with appraisal guidelines for studies using group designs in rehabilitation [20].

## 3 Results

**Fig. 1:**
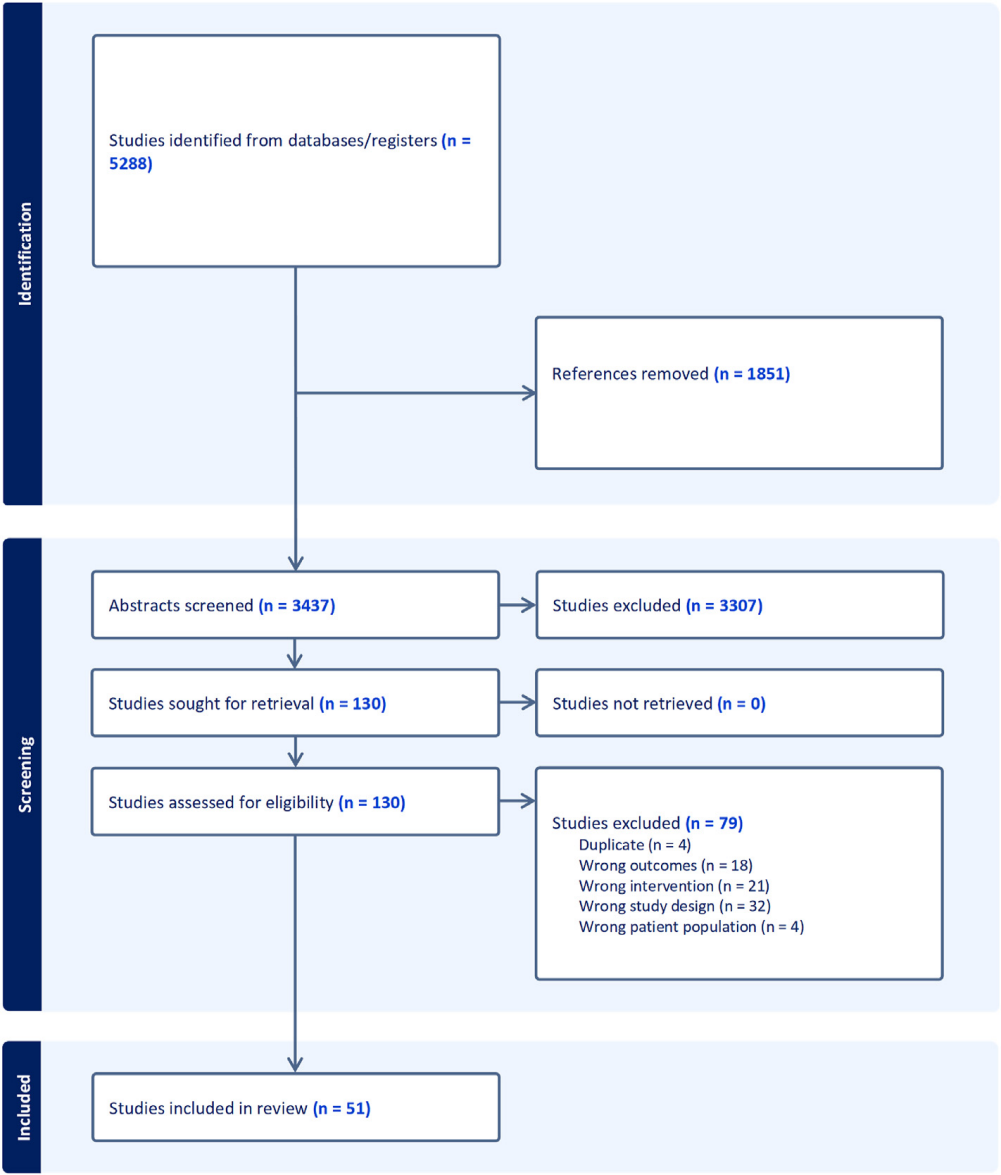
Prisma flow diagram.

The systematic search returned 5288 studies; 1816 duplicates were removed using EndNote, and a further 35 were removed using Covidence. A total of 3437 studies were screened through title and abstract (including 20 which were identified through citation searching of included studies), 130 of these met criteria for full-text screening. After full-text screening, 51 studies met eligibility criteria for inclusion and were appraised (see Fig. 1).

### 3.1 Participant characteristics

Characteristics of participants that completed exercise training interventions (N ​= ​836) are presented in Table 1. Children (33 ​%) and adolescents (52 ​%) comprised 85 ​% of participants and the iPPR of 3.27 met the criterion for gross overrepresentation. Adults (iPPR ​= ​0.22) and older adults (iPPR ​= ​0.00 ​%) were grossly underrepresented.

**Table 1:** Participant characteristics from 51 studies (N ​= ​836).

Participant characteristic	Participant sub-category	Participation in exercise training studies using group designs: N (Percent)	Prevalence in CP population – Percent	Indicative participation to prevalence ratio (iPPR)
**Age** (age prevalence from [29])	Middle childhood (6–12)	280 (33 ​%)	26.0 ​%	3.27^a^
	Adolescence (13–17)	435 (52 ​%)		
	Adults (18–64)	121 (14 ​%)	64.7 ​%	0.22^b^
	Older adults (>65)	1 (<1 ​%)	9.3 ​%	0.00^c^
	Not reported	0 (0 ​%)		
**Sex** (sex prevalence from [29])	Male	434 (52 ​%)	49.7 ​%	1.05
	Female	338 (40 ​%)	50.3 ​%	0.80
	Not reported	64 (8 ​%)	N/A	N/A
**Neurological subtype** (neurological subtype prevalence from [12])	Hypertonia (Incl. Quadriplegia, diplegia and hemiplegia)	750 (90 ​%)	80.1 ​%	1.12
	Dyskinesia (Incl. Dystonia and choreoathetosis)	10 (1 ​%)	5.6 ​%	0.18
	Ataxia	3 (<1 ​%)	4.0 ​%	0.10
	Mixed	14 (2 ​%)	1.3 ​%	1.53
	Not reported	57 (7 ​%)	N/A	N/A
**Functional effects** (GMFCS population prevalence estimate [30])	GMFCS I	294 (35 ​%)	34.2 ​%	1.07
	GMFCS II	190 (23 ​%)	25.6 ​%	0.92
	GMFCS III	85 (10 ​%)	11.5 ​%	0.91
	GMFCS IV	11 (1 ​%)	13.7 ​%	0.10
	GMFCS V	6 (<1 ​%)	15.6 ​%	0.00
	GMFCS I–IV	56 (7 ​%)	N/A	N/A
	GMFCS not reported	194 (23 ​%)	N/A	N/A
	MACS I	9 (1 ​%)	N/A	N/A
	MACS II	27 (3 ​%)	N/A	N/A
	MACS III	52 (6 ​%)	N/A	N/A
	MACS IV	3 (<1 ​%)	N/A	N/A
	MACS V	0 (0 ​%)	N/A	N/A
	MACS not reported	717 (89 ​%)	N/A	N/A
	BFMF I	5(<1 ​%)	N/A	N/A
	BFMF II	1 (<1 ​%)	N/A	N/A
	BFMF III	4 (<1 ​%)	N/A	N/A
	BFMF IV	1 (<1 ​%)	N/A	N/A
	BFMF V	1 (<1 ​%)	N/A	N/A
	BFMF not reported	794 (98 ​%)	N/A	N/A
	No studies reported eating or drinking function, vision function or communication function		N/A	N/A

GMFCS (Gross Motor Function Classification System; MACS (Manual Ability Classification System); BFMF (Bimanual Fine Motor Function).

a The numerator for this iPPR is 85 ​% and combines participation of the middle childhood category (31 ​%) with Adolescents (54 ​%), an age range of 6–17 years; the denominator (26 ​%) combines prevalence percentage from the 0–9yr (12.3 ​%) and 10–19yr (13.7 ​%), a total age range of 0–19yrs.

b The numerator for this iPPR is 15 ​% an age range of 18–65 years (adults); the denominator (64.7 ​%) combines the prevalence percentage from five age categories used in the source document – 20–29 years (14 ​%), 30–39 years (14.4 ​%), 40–49 years (14.5 ​%), 50–59 years (12.8 ​%) and 60–69 years (9.0 ​%), a total age range of 20–69 years.

c The numerator for this iPPR (<1 ​%) is the percentage of participants >65 years; the denominator (9.3 ​%) is the prevalence in the population of people with CP ​> ​70yrs.

Spastic hypertonia was the most commonly reported neurological subtype (90 ​%), consistent with population prevalence. Neurological subtypes that were grossly underrepresented in exercise training studies were dyskinetic (iPPR ​= ​0.18) and ataxic CP (iPPR ​= ​0.10). GMFCS Level was not specified for 23 ​% of participants. Where GMFCS was specified, study participation was commensurate with population prevalence for levels I, II and III. People with GMFCS level IV (iPPR ​= ​0.10) and V (iPPR ​= ​0.00) CP were grossly underrepresented.

### 3.2 Appraisal of prognostic variables

Studies reporting and accounting for prognostic variables (age, sex, neurological subtype, functional effects, and comorbidities); and inclusion/exclusion criteria are presented in Table 2.

**Table 2:** Appraisal of studies (N ​= ​51). Details presented are study design and participant characteristics (age, sex, neurological subtype, functional effects, and comorbidities). The table presents which prognostic variables are reported in each study (✓ or X) and whether or not sample heterogeneity in each prognostic variable is accounted for through trial conduct (✓ or X). Relevant exclusion criteria are also reported.

First author (Year)	Details	Prognostic variable appraisal										Exclusion criteria relating to: - Intervention safety/fidelity; - Competing interventions; and - Baseline PA level;
		Age		Sex		Neurological subtype		Functional effects		Comorbidities		
		Rep	Con	Rep	Con	Rep	Con	Rep	Con	Rep	Con	
Anderson (2003) [31]	**Design**: TG. **Age categories**: Adults**. Sex**: M&F. **Functional effects:** Combination of participants who are independently mobile, crutches, rollator, wheelchair, and 1 participant who uses a power chair. Others NR. **Neurological subtype**: NR. **Comorbidities:** NR. Statistical comparison of group characteristics performed at baseline.	✓	✓	✓	X	X	X	✓	X	X	X	**Intervention safety/fidelity**: N/A **Competing interventions:** N/A **Baseline PA level**: No participation in strength training in the past year.
Auld (2014) [32]	**Design**: SG. **Age categories**: Middle childhood & adolescence. **Sex**: M&F. **Functional effects:** GMFCS levels I & II. Others NR. **Neurological subtype**: Spastic diplegia and hemiplegia. **Comorbidities:** NR	✓	X	✓	X	✓	X	✓	✓	X	X	**Intervention safety/fidelity:** No intellectual or behavioural impairment impeding ability to follow verbal instructions; **competing interventions:** no current therapy program; no spasticity management, casting or orthopaedic surgery in past 6 months. **Baseline PA level:** N/A
Ballaz (2011) [33]	**Design**: SG. **Age categories**: Adolescents. **Sex**: NR. **Functional effects:** GMFCS level I, II, III and IV. Others NR. **Neurological subtype**: Spastic diplegia, hemiplegia or quadriplegia. **Comorbidities:** NR	✓	✓	✓	X	✓	X	✓	X	X	X	**Intervention safety/fidelity:** Able to follow simple verbal instructions; no cardiovascular disease; **competing interventions:** no recent (in past 8 months) surgical intervention or botulinum toxin a injection in the lower extremities. **Baseline PA level:** N/A.
Bania (2016) [34]	**Design**: TG. **Age categories**: Adolescents & adults. **Sex**: M&F. **Functional effects:** GMFCS II and III. Others NR. **Neurological subtype**: Bilateral spastic CP. **Comorbidities:** Hip pathology reported and accounted for, other comorbidities not reported.	✓	X	✓	X	✓	✓	✓	X	✓	✓	**Intervention safety/fidelity:** no contractures > 20° ​at the hip and knee; **competing interventions:** No single or multi-level orthopaedic surgery within the previous 2 years; **baseline PA level:** no resistance training in past 6 months.
Chen (2013) [35]	**Design**: TG. **Age categories**: Middle childhood. **Sex**: M&F. **Functional effects:** GMFCS levels I and II. Others NR. **Neurological subtype**: Spastic diplegic and hemiplegic CP. **Comorbidities:** NR Statistical comparison of groups performed at baseline.	✓	✓	✓	X	✓	X	✓	✓	X	X	**Intervention safety/fidelity:** poor cooperation or tolerance for testing; no recognized chromosomal abnormalities; progressive neurological disorder/severe concurrent illness or disease not typically associated with CP; active medical condition; hormonal disturbance; **competing interventions:** no major surgery or nerve block in the previous 3 months. **Baseline PA level:** N/A.
Cleary (2017) [36]	**Design**: TG. **Age categories**: Middle childhood & adolescence. **Sex**: M&F. **Functional effects:** GMFCS levels I, II and III. Others NR. **Neurological subtype**: NR. **Comorbidities:** NR	✓	X	✓	X	X	X	✓	X	X	X	**Intervention safety/fidelity:** had a reliable yes/no response; received medical clearance; **competing interventions:** no lower-limb surgery or botulinum toxin-A in past 6 months; **baseline PA level:** Not participated in an aerobic exercise program in past 6 months.
Clutterbuck (2022) [37]	**Design**: TG. **Age categories**: Middle childhood. **Sex**: M&F. **Functional effects:** GMFCS levels I & II; FMS50 and FMS500 also reported. **Neurological subtype**: Spastic and ‘other’ and motor distributions: Unilateral and bilateral. **Comorbidities:** Participants did not have medical co-morbidities impacting safe exercise as reported by their parents. Statistical comparison of group characteristics performed at baseline.	✓	✓	✓	X	✓	X	✓	✓	X	X	**Intervention safety/fidelity:** appropriate physical, behavioural and intellectual ability to complete assessments intervention; **competing interventions:** no orthopaedic or neurological surgery within six months; no botulinum toxin injections within three months prior to intervention; **baseline PA level:** N/A.
Colquitt (2020) [38]	**Design**: SG. **Age categories**: Middle childhood, adolescence & adulthood. **Sex**: M&F. **Functional effects:** BFMF levels I, II, III, IV and V; able to complete overhand throwing motion. Others NR. **Neurological subtype**: NR. **Comorbidities:** NR.	✓	X	✓	X	X	X	✓	X	X	X	**Intervention safety/fidelity:** Cognitive ability to follow directions; medically cleared for physical activity; **competing interventions:** no corrective surgery or botulinum toxin-A injections in past 6 months. **Baseline PA level:** N/A
Damiano (1998) [39]	**Design**: SG. **Age categories**: Middle childhood. **Sex**: NR. **Functional effects:** Required either hemiplegia that was at least a 20 ​% asymmetry in strength in two of the muscle groups tested; or spastic diplegia - moderately involved as determined by their status as a limited community ambulator and 50 ​% weakness from normal bilaterally in two of the lower extremity muscles tested. **Neurological subtype**: spastic diplegia and hemiplegia. **Comorbidities:** NR.	✓	✓	X	X	✓	X	✓	X	X	X	**Intervention safety/fidelity:** N/A; **competing interventions:** N/A; **baseline PA level:** N/A.
Damiano (1995) [40]	**Design**: TG. **Age categories**: Middle childhood. **Sex**: M&F. **Functional effects:** NR. **Neurological subtype**: Spastic diplegia. **Comorbidities:** NR. Control group were typically developing children.	✓	✓	✓	X	✓	✓	X	X	X	X	**Intervention safety/fidelity:** N/A; **competing interventions:** Prior orthopaedic surgery not an exclusion criterion if performed more than a year before participation; **baseline PA level:** N/A.
Darrah (1999) [41]	**Design**: SG. **Age categories**: Adolescents. **Sex**: M&F. **Functional effects:** Combination of preferred mode of mobility (independently mobile, walker, walking stick, wheelchair), other NR. **Neurological subtype**: Spastic hemiplegia, diplegia, quadriplegia, ataxia & dystonia. **Comorbidities:** NR.	✓	✓	✓	X	✓	X	✓	X	X	X	**Intervention safety/fidelity:** able to understand instructions; no severe physical or cognitive involvement; **competing interventions:** N/A; **baseline PA level:** N/A.
Dodd (2003) [42]	**Design**: TG. **Age categories**: Middle childhood & adolescents. **Sex**: M&F. **Functional effects:** GMFCS levels I, II and III, others NR. **Neurological subtype**: Spastic diplegia. **Comorbidities:** NR.	✓	X	✓	X	✓	✓	✓	X	X	X	**Intervention safety/fidelity:** able to follow simple commands; no fixed flexion deformity at the knee, hip >25°, fixed equinus >10°; **competing interventions:** no participation in serial casting, botulinum toxin, or orthopaedic surgery (less than 12 months); **baseline PA level**: no strength training within the previous 3 months.
Elnaggar (2019) [43]	**Design**: SG. **Age categories**: Middle childhood. **Sex**: M&F. **Functional effects:** GMFCS I & II. Others NR. **Neurological subtype**: Spastic hemiplegia. **Comorbidities:** NR Statistical comparison of group characteristics performed at baseline.	✓	✓	✓	X	✓	✓	✓	✓	X	X	**Intervention safety/fidelity:** able to understand and follow instructions; no cardiopulmonary disorders; no severe mental or physical co-morbidities that may result in activity limitation; **competing interventions:** no orthopaedic or neuromuscular surgery in last year; no neuromuscular blockers for tone management in last six months; **baseline PA level:** N/A.
Engsberg (2006) [44]	**Design**: SG. **Number:** 9. **Age categories**: Middle childhood & adolescence. **Sex**: M&F. **Functional effects:** GMFCS levels I, II and III; able to perform 6–8 reps of walking 9m, some ability to actively dorsiflex and plantarflex the foot; restricted hamstring length (90/90 test) to <45°; hypertonicity of the plantar flexors, no moderate-to-severe dystonia, athetosis, or ataxia; others NR. **Neurological subtype**: Spastic diplegia. **Comorbidities:** NR	✓	X	✓	X	✓	✓	✓	X	X	X	**Intervention safety/fidelity:** Cognitive skills to follow simple commands; **competing interventions:** no orthopaedic or neuromuscular surgery in last year; no Botox of casting in past 6 months; no surgical intervention in past year; no selective dorsal rhizotomy or intrathecal baclofen; **baseline PA level:** N/A.
Fosdahl (2019) [45]	**Design**: TG. **Age categories**: Middle childhood. **Sex**: M&F. **Functional effects:** GMFCS levels I, II, & III; <0° dorsal flexion in the ankle; <5° external rotation in the hips. Others NR. **Neurological subtype**: Bilateral spastic CP. **Comorbidities:** NR	✓	✓	✓	X	✓	✓	✓	X	X	X	**Intervention safety/fidelity:** able to cooperate or understand instructions; **competing interventions:** no surgical procedure in the lower limbs less than 1 year; no lower limb botulinum toxin a injections in past 6 months; **baseline PA level:** N/A.
Fowler (2010) [46]	**Design**: TG. **Age categories**: Middle childhood. **Sex**: M&F. **Functional effects:** GMFCS levels I, II, & III; good or fair selective voluntary motor control for at least one limb. Others NR. **Neurological subtype**: Spastic diplegia. **Comorbidities:** Reported - asthma, attention/behavioural problems, mental retardation, seizure disorder, learning problems, speech problems, vision problems, hearing problems. Statistical comparison of group characteristics performed at baseline.	✓	✓	✓	X	✓	✓	✓	X	✓	X	**Intervention safety/fidelity:** able to follow simple verbal directions and maintain age-appropriate behaviour; no serious medical conditions such as cardiac disease, diabetes, or uncontrolled seizures; no significant contractures; **competing interventions:** no surgery or baclofen pump implantation in past 12 months; no botulinum toxin injections, serial casting or orthoses in past 3 months; no initiation of oral neuromuscular medications in past 3 months; no initiation of physical therapy, exercise, sports activity, or change in assistive devices in past 3 months; **baseline PA level**: no current participation in a fitness program.
Fragala-Pinkham (2014) [47]	**Design**: SG. **Number:** 8. **Age categories**: Middle childhood & adolescents. **Sex**: M&F. **Functional effects:** GMFCS levels I and III. Others NR. **Neurological subtype**: Spastic diplegia and hemiplegia. **Comorbidities:** NR	✓	X	✓	X	✓	X	✓	X	X	X	**Intervention safety/fidelity:** able to follow directions; medically able to participate in an exercise program; no changes in medications or rehabilitation; no open wounds or swallowing precautions. **Competing interventions:** no history of botulinum toxin injections within 3 months or orthopaedic surgery within 6 months; **baseline PA level:** N/A.
Gibson (2018) [48]	**Design**: TG. **Number:** 21. **Age categories**: Middle childhood & adolescents. **Sex**: M&F. **Functional effects:** GMFCS levels I, II & III. Others NR. **Neurological subtype**: Spastic CP, one participant had spasticity and dystonia. **Comorbidities:** NR	✓	X	✓	X	✓	X	✓	X	X	X	**Intervention safety/fidelity:** able to understand assessment instructions; no cognitive or behavioural challenges that may interfere with group intervention; no medical condition that precluded participation in a vigorous exercise program; **competing interventions:** no surgery in past 6 months; **baseline PA level:** N/A.
Gillett (2018) [49]	**Design**: TG. **Age categories**: Adolescents and adults. **Sex**: M&F. **Functional effects:** GMFCS levels I & II; <-5° degrees ankle dorsiflexion in knee extension. Others NR. **Neurological subtype**: Spastic CP. **Comorbidities:** NR	✓	X	✓	X	✓	X	✓	✓	X	X	**Intervention safety/fidelity:** able to cooperate and understand instructions; **competing interventions:** no lower limb surgery in past 2 years; **baseline PA level:** no lower limb resistance training in past 6 months.
Hilderley (2020) [50]	**Design**: TG. **Number:** 20. **Age categories**: Middle childhood & adolescence. **Sex**: M&F. **Functional effects:** GMFCS levels I and II. Others NR. **Neurological subtype**: Spastic diplegic and hemiplegic CP. **Comorbidities:** NR	✓	X	✓	X	✓	X	✓	✓	X	X	**Intervention safety/fidelity:** able to follow multi-step instructions; **competing interventions:** no lower limb therapy during intervention; no surgery in past 9 months (muscle) or 12 months (bone); no botulinum toxin injections in lower limbs in past 4 months; **baseline PA level:** N/A.
Hjalmarsson [51]	**Design**: SG. **Number:** 15. **Age categories**: Middle childhood, adolescence & adulthood. **Sex**: M&F. **Functional effects:** GMFCS levels I, II, III and IV. Others NR. **Neurological subtype**: Spastic, dyskinetic or ataxic CP. Distribution NR. **Comorbidities:** NR	✓	X	✓	X	✓	X	✓	X	X	X	**Intervention safety/fidelity:** N/A; **competing interventions:** no orthopaedic surgery or injections of botulinum toxin in past three months; no interventions to reduce spasticity (e.g., selective dorsal rhizotomy or intrathecal baclofen); **baseline PA level:** N/A.
Hutzler (2013) [52]	**Design**: TG. **Number:** 10. **Age categories**: Adults. **Sex**: M&F. **Functional effects:** GMFCS levels II, III and IV; must score <45 on Barthel Index; have muscle tone and range of motion that allows the performance of manual movements with objects such as weights and bands. Others NR. **Neurological subtype**: NR. **Comorbidities:** NR	✓	✓	✓	X	X	X	✓	X	X	X	**Intervention safety/fidelity:** N/A; **competing interventions:** N/A; **baseline PA level:** N/A.
Hye-Jin (2020) [53]	**Design**: TG. **Age categories**: Middle childhood. **Sex**: M&F. **Functional effects:** GMFCS levels I II, and III. Others NR. **Neurological subtype**: Spastic diplegia. **Comorbidities:** NR	✓	✓	✓	X	✓	✓	✓	X	X	X	**Intervention safety/fidelity:** able to follow instructions; no unstable seizures; no other diseases that interfered with physical activity; **competing interventions:** no botulinum toxin type a injections in past 3 months; no surgery in past 6 months; **baseline PA level:** N/A.
Izadi (2006) [54]	**Design**: TG. **Age categories**: Middle childhood & adolescence. **Sex**: NR. **Functional effects:** NR. **Neurological subtype**: Spastic diplegia. **Comorbidities:** NR	✓	X	X	X	✓	✓	X	X	X	X	**Intervention safety/fidelity:** N/A; **competing interventions:** N/A; **baseline PA level:** N/A.
Kalkman (2019) [55]	**Design**: TG. **Age categories**: Middle childhood & adolescence. **Sex**: M&F. **Functional effects:** GMFCS levels I, II and III; able to perform at least 1 bilateral heel raise. Others NR. **Neurological subtype**: Spastic hemiplegia and diplegia. **Comorbidities:** NR	✓	X	✓	X	✓	X	✓	X	X	X	**Intervention safety/fidelity:** N/A; **competing interventions:** no botulinum toxin-A injections to the lower limb in past 6 months; no baclofen pump; no lower limb neuro- or orthopaedic surgery; **baseline PA level:** N/A.
Kaya Kara (2019) [56]	**Design**: TG. **Age categories**: Middle childhood & adolescence. **Sex**: M&F. **Functional effects:** GMFCS levels I; MACS levels I, II and III. Others NR. **Neurological subtype**: Spastic hemiplegia. **Comorbidities:** NR	✓	X	✓	X	✓	✓	✓	X	X	X	**Intervention safety/fidelity:** able to follow verbal instructions; **competing interventions:** no orthopaedic surgery or botulinum toxin injection in past 6 months, no epilepsy, no other disease that interfered with physical activity; **baseline PA level:** N/A.
Kirk (2016) [57]	**Design**: TG. **Age categories**: Adults. **Sex**: M&F. **Functional effects:** GMFCS levels I, II and III comprising a range of reported functional mobility: Independently mobile, crutches, rollator, wheelchair, and power chair. Others NR. **Neurological subtype**: Spastic diplegia, hemiplegia and quadriplegia. **Comorbidities:** Medications, surgical history are reported.	✓	✓	✓	X	✓	X	✓	X	✓	X	**Intervention safety/fidelity:** Ability to speak or read; no other severe chronic diseases; no pregnancy; **competing interventions:** N/A; **baseline PA level:** All subjects had previous experience with resistance training, but never with such heavy resistance and never with a focus on the explosive execution of the exercises.
Kruse (2019) [58]	**Design**: TG. **Age categories**: Middle childhood & adolescence. **Sex**: M&F. **Functional effects:** GMFCS levels I & II. Others NR. **Neurological subtype**: Spastic hemiplegia & diplegia. **Comorbidities:** NR	✓	X	✓	X	✓	X	✓	✓	X	X	**Intervention safety/fidelity:** able to follow verbal instructions; **competing interventions:** no previous surgery to the plantar flexors or botulinum toxin) in the past 6 months; **baseline PA level:** N/A.
Lee (2015) [59]	**Design**: TG. **Age categories**: Middle childhood. **Sex**: M&F. **Functional effects:** GMFCS levels I, II & III. Others NR. **Neurological subtype**: Spastic, distribution NR. **Comorbidities:** NR	✓	✓	✓	X	X	X	✓	X	X	X	**Intervention safety/fidelity:** able to follow instructions; no unstable seizures; no other disease that would interfere with physical activity; **competing interventions:** no treatment for spasticity or surgery in past 6 months; no change in medication during study; **baseline PA level:** N/A.
Liao (2007) [60]	**Design**: TG. **Age categories**: Middle childhood. **Sex**: M&F. **Functional effects:** GMFCS levels I & II; no obvious limitation in the passive range of motion of lower extremities. Others NR. **Neurological subtype**: Spastic diplegia. **Comorbidities:** NR	✓	✓	✓	X	✓	✓	✓	✓	X	X	**Intervention safety/fidelity:** able to follow verbal instructions; no orthopaedic problems or medical conditions that prevented participation; **competing interventions:** no orthopaedic intervention, selective dorsal rhizotomy, or botulinum toxin injection to the lower extremities within 6 months; **baseline PA level:** no strength training in past 3 months.
MacPhail (1995) [61]	**Design**: SG. **Age categories**: Adolescence. **Sex**: M&F. **Functional effects:** NR. **Neurological subtype**: Hemiplegia, diplegia or quadriplegia. **Comorbidities:** NR	✓	✓	✓	X	✓	X	X	X	X	X	**Intervention safety/fidelity:** Adequate cognitive ability and strength, co-ordination and knee-extension range of motion (KOM) to operate the isokinetic dynamometer; **competing interventions:** N/A; **baseline PA level:** N/A.
Maeland (2009) [62]	**Design**: TG. **Age categories**: Adults. **Sex**: M&F. **Functional effects:** GMFCS levels II & III comprising a range of reported functional mobility: Independently mobile, orthopaedic insole, stick, wheelchair, and power chair. Others NR. **Neurological subtype**: Spastic diplegia; one participant also had dyskinesia. **Comorbidities: NR**	✓	✓	✓	X	✓	✓	✓	X	X	X	**Intervention safety/fidelity:** no severe cognitive disorders; **competing interventions:** N/A; **baseline PA level:** No strength training for the lower limbs during the past year.
McCubbin (1985) [63]	**Design**: TG. **Age categories**: Adolescents and adults. **Sex**: NR. **Functional effects:** Classified using the 10-class National association of sport for cerebral palsy (NASCP) system (participant class range 1–8). Others NR. **Neurological subtype**: Spastic, athetoid and mixed CP, distribution NR. **Comorbidities:** NR	✓	X	X	X	✓	X	✓	X	X	X	**Intervention safety/fidelity:** N/A; **competing interventions:** N/A; **baseline PA level:** N/A.
Mitchell (2016) [64]	**Design**: TG. **Age categories**: Middle childhood & adolescence. **Sex**: M&F. **Functional effects:** GMFCS levels I & II; MACS levels I, II and III; no obvious limitation in the passive range of motion of lower extremities. Others NR. **Neurological subtype**: Spastic unilateral CP. **Comorbidities:** Reported - intellectual impairment, learning difficulties, ASD, ADHD, visual impairment, hearing impairment, epilepsy.	✓	X	✓	X	✓	✓	✓	X	✓	X	**Intervention safety/fidelity:** no unstable epilepsy or medical conditions that would preclude participation in training; **competing interventions:** no upper-limb botulinum toxin a injections or surgery in the previous 2 months or 6 months respectively; **baseline PA level:** N/A.
Moreau (2013) [65]	**Design**: TG. **Age categories**: Middle childhood & adolescence. **Sex**: M&F. **Functional effects:** GMFCS levels I, II & III. Others NR. **Neurological subtype**: Spastic hemiplegia & diplegia. **Comorbidities:** NR.	✓	X	✓	X	✓	X	✓	X	X	X	**Intervention safety/fidelity:** able to follow and understand commands; **competing interventions:** no orthopaedic or neurosurgery in past year, no botulinum toxin injections in past 4 months; **baseline PA level:** N/A.
Morton (2005) [66]	**Design**: SG. **Age categories**: Middle childhood. **Sex**: M&F. **Functional effects:** GMFCS level III. Choice of walking aid variable: Kaye-walker, quadruped sticks, elbow crutches. Others NR. **Neurological subtype**: Bilateral spastic CP. **Comorbidities:** NR	✓	✓	✓	X	✓	✓	✓	✓	X	X	**Intervention safety/fidelity:** able to follow instructions; no debilitating illness before or during study; no cardiac or respiratory condition affecting exercise; **competing interventions:** no surgery or orthopaedic procedures in past 6 months; no medication changes; **baseline PA level:** N/A.
Nsenga (2013) [67]	**Design**: TG. **Age categories**: Middle childhood & adolescence. **Sex**: M&F. **Functional effects:** GMFCS levels I & II. Others NR. **Neurological subtype**: NR. **Comorbidities:** Reported - chest wall deformity.	✓	X	✓	X	X	X	✓	✓	✓	X	**Intervention safety/fidelity:** no cardiac or respiratory conditions that could be negatively affected by exercise; **competing interventions:** no surgery or orthopaedic procedures in past 6 months; no orthopaedic treatment, neurosurgery or botulinum toxin injection in past 6 months; **baseline PA level:** N/A.
Peungsuwan (2017) [68]	**Design**: TG. **Age categories**: Middle childhood & adolescence. **Sex**: M&F. **Functional effects:** GMFCS levels I, II & III with a range of reported functional mobility: Independently mobile, crutches, wheeled walker, walker. Others NR. **Neurological subtype**: Spastic hemiplegia & diplegia. **Comorbidities:** NR.	✓	X	✓	X	✓	X	✓	X	X	X	**Intervention safety/fidelity:** able to understand verbal instructions; no serious medical conditions which contraindicated exercise; no lower limb muscle contractures; **competing interventions:** no botulinum toxin injections or surgical procedures in past 3 months; **baseline PA level:** no other exercise training in past 4 months.
Reid (2010) [69]	**Design**: TG. **Age categories**: Middle childhood & adolescence. **Sex**: M&F. **Functional effects:** MACS levels I, II & III; others NR. **Neurological subtype**: 13 participants had spastic hemiplegia; one had spastic triplegia. **Comorbidities:** NR.	✓	X	✓	X	✓	✓	✓	X	X	X	**Intervention safety/fidelity:** able to follow two-step instructions; **competing interventions:** no previous upper-limb surgery or pharmacological treatment for spasticity (botulinum toxin A) in past 12 months **baseline PA level:** no upper-limb strength training in past 12 months.
Ryan (2020) [70]	**Design**: TG. **Age categories**: Middle childhood & adolescence. **Sex**: M&F. **Functional effects:** GMFCS levels I, II & III; able to activate plantar flexors. Others NR. **Neurological subtype**: Unilateral & bilateral spastic CP. **Comorbidities:** NR.	✓	X	✓	X	✓	✓	✓	✓	X	X	**Intervention safety/fidelity:** Cognitively able to comply with assessment and training. **Competing interventions:** no orthopaedic surgery in lower limbs in past 12 months; no botulinum neurotoxin a injections or serial casting in past 6 months; **baseline PA level:** N/A.
Scholtes (2010) [71]	**Design**: TG. **Age categories**: Middle childhood. **Sex**: M&F. **Functional effects:** GMFCS levels I, II & III. Others NR. **Neurological subtype**: Unilateral & bilateral spastic CP. **Comorbidities:** Puberty onset derived from the Tanner stages of sexual maturation; problem behaviour also reported. Other functional effects and comorbidities not reported. Statistical comparison of group characteristics performed at baseline.	✓	✓	✓	X	✓	X	✓	X	✓	X	**Intervention safety/fidelity:** able to follow verbal instructions and participate in a group training; no unstable seizures; no other diseases that interfered with physical activity; **competing interventions:** no treatment for spasticity or surgical procedures in past 3 months (for botulinum toxin type a injections) or 6 months (for surgery); no any change in medication during the study; **baseline PA level:** N/A.
Shinohara (2002) [72]	**Design**: TG. **Age categories**: Adolescents. **Sex**: NR. **Functional effects:** NR. **Neurological subtype**: Spastic CP, distribution NR. **Comorbidities:** NR	✓	✓	X	X	✓	X	X	X	X	X	**Intervention safety/fidelity:** N/A; **competing interventions:** N/A; **baseline PA level:** N/A.
Taylor (2004) [73]	**Design**: SG. **Age categories**: Adults. **Sex**: M&F. **Functional effects:** Range of preferred mode of mobility reported: Independently mobile, sticks, pick-up frame, manual wheelchair and electric wheelchair. All participants had high support needs (assessed via Barthel ADL Index). Others NR. **Neurological subtype**: Spastic diplegia, hemiplegia and athetoid CP. **Comorbidities:** Reported - cognitive impairment, hip subluxation, osteoarthritis, joint contractures.	✓	✓	✓	X	✓	X	✓	X	✓	X	**Intervention safety/fidelity:** able to clearly communicate (by any means including the use of augmentative communication devices); be self-advocating (the ability to be able to communicate whether they wanted to participate or not); **competing interventions:** N/A; **baseline PA level:** no strength-training in the past 3 months.
Taylor (2013) [74]	**Design**: TG. **Age categories**: Adolescents and adults. **Sex**: Males and females. **Functional effects:** GMFCS levels II & III, range of preferred mode of mobility reported: Independently mobile, sticks, crutches, and walkers. Others NR. **Neurological subtype**: Spastic diplegia. **Comorbidities:** Reported - hip morphology.	✓	X	✓	X	✓	✓	✓	X	✓	X	**Intervention safety/fidelity:** able to follow simple instructions; no contractures of more than 20° at the hip and knees; **competing interventions:** no single event or multi-level orthopaedic surgery in the past 2 years; **baseline PA level:** no strength training programme in past 6 months.
Tedla (2014) [75]	**Design**: TG. **Age categories**: Middle childhood & adolescence. **Sex**: M&F. **Functional effects:** GMFCS levels I, II, III & IV; ability to sit unsupported for 10 ​s with feet supported, ability to move lower extremities in gravity-eliminated positions. Others NR. **Neurological subtype**: Spastic diplegia. **Comorbidities:** NR.	✓	X	✓	X	✓	✓	✓	X	X	X	**Intervention safety/fidelity:** Minimum score required on modified mini mental state examination; no cardiorespiratory or cardiac conditions affected by exercise; no seizures in past 1 years; no debilitating disease; no flexion deformity at hip or knee >25°; **competing interventions:** no orthopaedic surgery in lower limbs in past 12 months; no botulinum toxin a injections in past 6 months; no selective dorsal rhizotomy; no medications to alter muscle strength or tone; **baseline PA level:** no strength training in past 3 months.
Terada (2017) [76]	**Design**: SG. **Age categories**: Adults. **Sex**: M&F. **Functional effects:** GMFCS level V. Others NR. **Neurological subtype**: Severe athetospastic CP. **Comorbidities:** NR	✓	✓	✓	X	✓	✓	✓	✓	X	X	**Intervention safety/fidelity:** no severe difficulty of communication; no history of cardiorespiratory disease; **competing interventions:** no medications that could affect results (e.g., β-blockers); no surgery in the past year; **baseline PA level:** no prior experience with sport.
Unnithan (2007) [77]	**Design**: TG. **Age categories**: Adolescents. **Sex**: M&F. **Functional effects:** All participants able to walk, however a range of preferred mode of mobility: Independently mobile, anterior walker and wheelchair. Others NR. **Neurological subtype**: Spastic diplegia. **Comorbidities:** NR	✓	✓	✓	X	✓	✓	✓	X	X	X	**Intervention safety/fidelity:** N/A; **competing interventions:** no orthopaedic surgical operation or botulinum toxin injections for the treatment of spasticity in past 1 year; **baseline PA level:** no recent engagement in systematic exercise.
Uysal (2024) [78]	**Design**: TG. **Age categories**: Children. **Sex**: M&F. **Functional effects:** GMFCS levels I & II. Others NR. **Neurological subtype**: Monoplegic, hemiplegic or diplegic. **Comorbidities:** NR	✓	✓	✓	X	✓	X	✓	✓	X	X	**Intervention safety/fidelity:** no hearing and visual impairment; no secondary orthopaedic problems; no cognitive impairment. **Competing interventions:** no surgery in the last 6 months. **Baseline PA level:** N/A.
Van den Berg-Emons (1998) [79]	**Design**: TG. **Age categories**: Middle childhood. **Sex**: M&F. **Functional effects:** Ambulant and non-ambulant (wheelchair users). Others NR. **Neurological subtype**: Spastic CP; two participants also had ataxia. Distribution NR. **Comorbidities:** NR	✓	✓	✓	X	✓	✓	✓	X	X	X	**Intervention safety/fidelity:** N/A; **competing interventions:** N/A; **baseline PA level:** N/A.
Van Wely (2014) [80]	**Design**: TG. **Age categories**: Middle childhood. **Sex**: M&F. **Functional effects:** GMFCS levels I, II and III. Others NR. **Neurological subtype**: Unilateral and bilateral spastic CP; not predominantly dyskinetic or ataxic movement disorder. **Comorbidities:** NR	✓	✓	✓	X	✓	X	✓	X	X	X	**Intervention safety/fidelity:** Understanding of the Dutch language; no unstable seizures, no contraindications for physical training, no severe behavioural problems or severe intellectual disability; **competing interventions:** no surgery in past 6 months, no botulinum toxin treatment or serial casting in past 3 months; **baseline PA level:** no regular participation in sports or fitness program.
Verschuren (2007) [81]	**Design**: TG. **Age categories**: Middle childhood & adolescence. **Sex**: M&F. **Functional effects:** GMFCS levels I & II. **Neurological subtype**: Unilateral and bilateral spastic CP. **Comorbidities:** NR	✓	X	✓	X	✓	X	✓	✓	X	X	**Intervention safety/fidelity:** no cardiac or respiratory conditions that could negatively be affected by exercise; **competing interventions:** No orthopaedic surgery or neurosurgery and/or botulinum toxin injection(s) in past 6 months; **baseline PA level:** N/A.

TG ​= ​two-group; SG ​= ​single-group; M ​= ​Male; F = Female; Rep ​= ​Reported; NR = Not reported; Con ​= ​Accounted for through trial conduct; PA ​= ​physical activity; GMFCS ​= ​Gross Motor Function Classification System; MACS ​= ​Manual Ability Classification System; BFMF = Bimanual Fine Motor Function.

#### 3.2.1 Age

All 51 studies reported participant age. Twenty-six studies accounted for the effects of age and included participants only in either middle childhood (n ​= ​15, 29 ​%), adolescence (n ​= ​5, 10 ​%) or adulthood (n ​= ​6, 12 ​%). The remaining 25 studies did not account for the effects of age. The populations of these studies comprised participants in a combination of either middle childhood and adolescence (n ​= ​19, 38 ​%), adolescence and adulthood (n ​= ​4, 8 ​%) or middle childhood, adolescence and adulthood (n ​= ​2, 4 ​%).

#### 3.2.2 Sex

Participant sex was reported in forty-seven of the 51 studies (92 ​%). In all studies where sex was reported, the study population comprised a combination of males and females, therefore no studies accounted for the effects of participant sex.

#### 3.2.3 Neurological subtype

Forty-five of the 51 studies (88 ​%) reported participant neurological subtype. Twenty-one studies (41 ​%) reported and accounted for the effects of neurological subtype and included participants with only either spastic diplegia (n ​= ​17), spastic hemiplegia (n ​= ​3) or spastic athetoid (n ​= ​1). Twenty-four studies (47 ​%) reported but did not account for the effects of neurological subtype. The populations of these studies comprised participants in a combination of either spastic monoplegia, diplegia and hemiplegia (n ​= ​14), or spastic CP combined with dystonia, ataxia or ‘other’ neurological subtypes (n ​= ​10).

#### 3.2.4 Functional effects

Forty-six of the 51 studies (90 ​%) reported participant functional effects. Thirty-three studies (65 ​%) reported GMFCS only, 2 studies reported GMFCS and MACS and 2 studies reported GMFCS and other measures of physical function (e.g., FMS50, FMS500, Barthel Index). Four studies used measures of physical function other than GMFCS. The remaining 5 studies (10 ​%) provided non-standardised descriptions of physical function. No studies reported EDACS, VFCS or CFCS.

Fourteen studies (27 ​%) reported and accounted for the effects of GMFCS Level and included participants only at either GMFCS Levels I and II (n ​= ​12), III (n ​= ​1) or Level V (n ​= ​1). This includes one study (Ryan et al., 2020) in which participants were GMFCS I-III with unilateral or bilateral spastic CP, but additional statistical analysis was conducted to determine whether treatment effect differed according to GMFCS level or motor distribution, and to account for major imbalances in prognostic factors between groups. One study included participants at GMFCS Level I only, but MACS Levels I-III; and one study included participants at GMFCS Levels I and II only, but MACS Levels I-III. Thirty-three studies (65 ​%) reported but did not account for functional effects.

#### 3.2.5 Comorbidities

Eight of the 51 studies (16 ​%) reported comorbidities. Across the 8 studies the comorbidities reported included seizure disorders and physical (e.g., hip subluxation, joint contractures, osteoarthritis), behavioural (e.g., Autism Spectrum Disorder, Attention Deficit Hyperactivity Disorder), cognitive/intellectual, or sensory (hearing, vision) impairments. Only one study accounted for the effects of hip pathology.

#### 3.2.6 Exclusion criteria

Forty-five of the 51 studies (88 ​%) reported exclusion criteria. Seventeen studies (33 ​%) enhanced sample homogeneity by excluding based on training history (i.e., engaged in similar training previously [n ​= ​17]). Thirty-nine of the 51 studies (76 ​%) excluded participants receiving other medical or therapeutic interventions (e.g., surgery [n ​= ​38], botulinum toxin A injection [n ​= ​29], or serial casting [n ​= ​7]). Thirty-eight of the 51 studies (74 ​%) excluded for safety/fidelity reasons (e.g., unable to follow testing/training instructions [n ​= ​36], or medical risk [n ​= ​20]).

## 4 Discussion

This study appraised the representativeness and homogeneity of samples in exercise training studies using group designs in people with CP. Findings indicate that children and adolescents are over-represented in the literature, and adults, wheelchair users, people with dyskinetic or ataxic CP and certain comorbidities are grossly under-represented. The majority of the studies identified in this paper did not account for the range of individual and clinical prognostic variables known to impact exercise training responses in this population. Below we discuss the implications of these findings in relation to sample representativeness and homogeneity. We also consider challenges and future directions for research.

### 4.1 Sample representativeness

Results of the iPPR analysis indicate that researchers have focused disproportionately on children and adolescents (prevalence ​= ​26.0 ​%; iPPR ​= ​3.27), particularly those with spastic hypertonia who walk (GMFCS I, II and some III's) – see Table 1. Adults, who comprise more than 60 ​% of the CP population, are grossly underrepresented in exercise trials using group designs (iPPR ​= ​0.22) despite increasing evidence of their complex health needs and high prevalence of pain and fatigue [16,21,22].

Other sub-populations that are grossly underrepresented are older adults (prevalence ​= ​9.3 ​%; iPPR ​= ​0.00), people with dyskinesia (prevalence ​= ​5.6 ​%; iPPR ​= ​0.18) and ataxia (prevalence ​= ​4.0 ​%; iPPR ​= ​0.10) and those who use wheelchairs – GMFCS IV (prevalence ​= ​13.7 ​%; iPPR ​= ​0.10) and V (prevalence ​= ​15.6 ​%; iPPR ​= ​0.00). While previous reviews have noted some of these imbalances [[5], [6], [7], [8]], appraisals have not been benchmarked and therefore the scale of neglect has been difficult to quantify.

The research community should direct greater attention and resources to understanding exercise training responses and developing effective exercise interventions for neglected CP subpopulations. In the meantime, reviews of evidence from exercise training studies in people with CP should formally evaluate and report the iPPR for key subpopulations and report any imbalances explicitly and prominently. Conclusions reached or recommendations made should be qualified based on the representativeness of the data. Further, practitioners who rely on the scientific record to inform practice should also appropriately downgrade the strength of any exercise training advice to patients from neglected subpopulations.

### 4.2 Sample homogeneity

Our appraisal of sample homogeneity in the 51 included studies (see Table 2) revealed that exclusion criteria which enhanced sample homogeneity, did so at the expense of participants with common co-morbidities. For example, 72 ​% of studies explicitly excluded people who could not follow required instructions, a criterion that will exclude many of the estimated 50 ​% of people with CP who have an intellectual disability and 25 ​% with a behavioural disorder such as ADHD or autism. We found no studies which evaluated interventions developed specifically for people with CP who have intellectual or behavioural impairments. Given their high prevalence, and the inherent importance of long-term adherence to exercise, there is clearly a need for such studies.

The majority of the studies identified in this paper did not account for the range of individual and clinical prognostic variables known to impact exercise in this population. Of the included studies, 92 ​% reported participant sex, but 100 ​% of those studies treated the results from males and females with CP as an undifferentiated whole. This is despite well documented sex-based differences in exercise training responses [3,23].

In regard to age, 37 ​% of studies made the unsupported assumption that exercise training responses for pre-pubescent children (6–12yrs) and pubescent adolescents (13–17 years) would be equivalent. This assumption was compounded by the universal treatment of males and females as a single group because sex-based differences in exercise training responses increase markedly during adolescence. For example, between 11 and 16 years of age, fat-free mass increases by 40 ​% in girls but 90 ​% in boys, driving significant sex-based divergence in both peak oxygen uptake (10 ​% greater in prepubescent boys but 40 ​% greater post-pubescent boys) and muscular strength [24]. Furthermore, gross motor function of people with CP improves during childhood but then during adolescence either plateaus (GMFCS I, II) or declines (GMFCS III-V) [25]. Children and adolescents with CP should be treated separately for the purposes of evaluating exercise training responses.

Fifteen studies (29 ​%) restricted study participants to children, the life-stage when sex-based differences are minimised. However the benefits of such design rigour are negated if systematic reviews pool results from studies of male and female children and adolescents, as four recent reviews have done [[5], [6], [7], [8]].

Like random allocation of participants, sample homogeneity is a research design feature that safeguards the internal validity of research studies using group designs. Both features act to reduce noise in the data and optimise the signal-to-noise ratio. In this review, 38 studies with more than one group were identified and, although 31 of these studies (82 ​%) randomised participants, our results indicate that the participants in these studies varied widely in relation to key prognostic variables such as age, sex and functioning. The result of this heterogeneity is predictable, systematic between-participant differences in exercise training responses which act to amplify noise and reduce internal validity [4,17]. The reduction is independent of randomisation.

### 4.3 Challenges

Studies using group designs present researchers in this field with two competing interests that are particularly difficult to reconcile – achieving sufficient sample size and achieving sufficient sample homogeneity. Our review demonstrates that to date, sample homogeneity has received little priority and that therefore evidence quality is likely to be lower than previously thought.

It is possible that the lack of emphasis on sample homogeneity in research to date has been legitimised to some degree by research quality appraisal tools. A recent review of tools used to assess the risk of bias and reporting quality of randomised controlled trials in rehabilitation identified 11 tools that included 6 items related to the appraisal of participant selection for an RCT [20]. One of the 6 items related to ensuring sample homogeneity [20] however only one of the 11 tools reviewed included this item – the Maastricht Tool [26], published in 1997. Tools commonly used to appraise the quality of CP research studies such as Pedro and RoB 2.0 did not include this item, giving the misleading impression that sample homogeneity is not important.

### 4.4 Future directions

Overall, in order to better understand how people with CP respond to exercise training interventions we are in favour of a more nuanced and differentiated approach. This approach should use research designs which prioritise internal validity and which take into account the heterogeneity of the prognostic variables which are a defining feature of the CP population, as well as the uneven distribution of prognostic variables in the CP population.

In our assessment, there might be certain CP subpopulations that lend themselves to group level research designs. For example, a study of exercise training responses in high functioning (GMFCS I and II) prepubescent children (6–12 years), with spastic diplegic CP who do not have intellectual or behavioural impairments could reach a relatively defensible level of homogeneity and results of our review indicate that researchers have relatively good access to this population. However, the reporting standards for establishing sample homogeneity should be much stricter than those currently in place. Specifically, instead of reporting group level prognostic variables – the mean age, standard deviation and range of each group; the number of people at GMFCS Level I and Level II in each group and the number and type of comorbidities in each group – studies should provide supplementary material which describes each study participant in relation to the key prognostic variables. Additionally, each individual participants’ response to the exercise intervention or the control condition as measured using the key outcomes should be presented in a waterfall plot which permits matching of individual results with individual clinical profiles.

Researchers may also wish to empirically test whether, and to what extent, different CP subpopulations do respond differently to the same intervention. For example, a comparative study of lower-limb strength training responses in prepubescent and adolescent males with spastic CP, GMFCS I and II might provide a basis for developing a method of statistical adjustment that would subsequently permit studies to combine populations.

However, we believe that group research designs are not an appropriate or practical means of addressing the need for studies evaluating exercise training responses in many under-researched subsections of the CP population. This includes, but is not limited to: people with CP who use wheelchairs – GMFCS III, IV and V – because of their more severe neurological involvement, their higher rate and severity of comorbidities and the significantly elevated time-cost associated with being physically active [27]; people with dyskinetic and ataxic cerebral palsy because of their low incidence, unique nature and higher rate of comorbidities; and people with CP who have intellectual and/or behavioural impairments. A far greater emphasis should be placed on single-case experimental designs (SCEDs) in which each participant acts as their own control. SCEDs are recognised to generate high-level evidence while also conferring a range of other important advantages [28] including: permitting much closer supervision of people with multiple comorbidities who are at increased risk of adverse events (e.g. exercise induced seizures); more capacity to individualise and to provide personal assistance; provide opportunity for longer follow-up/longitudinal study; but most importantly, overcome the arguably impossible task of achieving both adequate sample size and satisfactory sample homogeneity in relation to key prognostic variables in group level studies.

Finally, systematic reviews should not be restricted to studies that employ group designs or RCTs. Greater and more coordinated efforts to conduct high-quality SCEDs of exercise training interventions would create the possibility of aggregating results from SCEDs to generate more specific and greatly needed new knowledge in understudied populations. Appropriately qualified consideration should also be given to evidence from studies employing designs which generate lower quality evidence in otherwise understudied populations.

## 5 Conclusion

This review demonstrates that, to date, large portions of the CP subpopulation are grossly under-represented in exercise training studies using group designs and that samples are highly heterogeneous in relation to key prognostic variables. This means little is known about underrepresented CP subpopulations and that, overall, the quality of evidence in the field is probably lower than previously thought. It is possible these findings may be true of other studies evaluating other therapeutic interventions for people with CP. In the interests of ensuring all people with CP benefit from scientific advances, similar appraisals are required in those fields.

## Author contributions

S.M. Tweedy PhD was the lead investigator. He oversaw and contributed to all aspects of the project.

I. M. Dutia contributed to project design, search and screening, analysis and manuscript preparation.

L. Caughey completed an initial honors project on this study, and contributed to project design, search and screening and analysis.

B. Demetriou also assisted with project design, search and screening and analysis.

E. M. Beckman contributed to project design, and intellectually reviewed the manuscript.

J. Cairney PhD provided intellectual oversight of the project, protocol and provided substantial contributions to the manuscript.

## Data sharing statement

Data will be shared upon reasonable request.

## Declaration of competing interest

None.

## Appendix 1 Search terms

### **PubMed:**

((cerebral palsy[MeSH Terms]) OR ("Cerebral Palsy"[Title/Abstract] OR athetoid[Title/Abstract] OR ataxic[Title/Abstract] OR athetosis[Title/Abstract] OR dyskin∗[Title/Abstract] OR dyston∗[Title/Abstract] OR "choreo-athetotic"[Title/Abstract] OR spastic[Title/Abstract] OR hypertonia[Title/Abstract]))

AND.

((exercise[MeSH Terms]) OR (Exercis∗[Title/Abstract] OR "physical activity"[Title/Abstract] OR training[Title/Abstract] OR fitness[Title/Abstract] OR sport[Title/Abstract] OR strength[Title/Abstract] OR resistance[Title/Abstract] OR running[Title/Abstract] OR "race run∗"[Title/Abstract] OR racerun∗[Title/Abstract] OR walk∗[Title/Abstract] OR cycle[Title/Abstract] OR cycling[Title/Abstract] OR bicycl∗[Title/Abstract] OR bike[Title/Abstract] OR rowing[Title/Abstract] OR aerobic[Title/Abstract] OR anaerobic[Title/Abstract] OR endurance[Title/Abstract] OR swim∗[Title/Abstract] OR aquatic∗[Title/Abstract] OR dance[Title/Abstract] OR dancing[Title/Abstract] OR yoga[Title/Abstract] OR "tai chi"[Title/Abstract] OR Pilates[Title/Abstract] OR "para sport"[Title/Abstract] OR "active video gaming"[Title/Abstract] OR exergam∗[Title/Abstract]))

AND.

Filters: 1980, clinical trial, humans and English.

### **CINAHL:**

(((TI "Cerebral Palsy" OR AB "Cerebral Palsy") OR (TI athetoid OR AB athetoid) OR (TI ataxic OR AB ataxic) OR (TI athetosis OR AB athetosis) OR (TI dyskin∗ OR AB dyskin∗) OR (TI dyston∗ OR AB dyston∗) OR (TI choreo-athetotic OR AB choreo-athetotic) OR (TI spastic OR AB spastic) OR (TI hypertonia OR AB hypertonia))) AND (((MH exercise+)) OR ((TI Exercis∗ OR AB Exercis∗) OR (TI "physical activity" OR AB "physical activity") OR (TI training OR AB training) OR (TI fitness OR AB fitness) OR (TI sport OR AB sport) OR (TI strength OR AB strength) OR (TI resistance OR AB resistance) OR (TI running OR AB running) OR (TI "race run∗" OR AB "race run∗") OR (TI racerun∗ OR AB racerun∗) OR (TI walk∗ OR AB walk∗) OR (TI cycle OR AB cycle) OR (TI cycling OR AB cycling) OR (TI bicycl∗ OR AB bicycl∗) OR (TI bike OR AB bike) OR … AND (randomized controlled trials OR MH double-blind studies OR MH single-blind studies OR MH random assignment OR MH pretest-posttest design OR MH cluster sample OR TI (randomised OR randomized) OR AB (random∗) OR TI (trial) OR (MH (sample size) AND AB (assigned OR allocated OR control)) OR MH (placebos) OR PT (randomized controlled trial) OR AB (control W5 group) OR MH (crossover design) OR MH (comparative studies) OR AB (cluster W3 RCT)) NOT ((MH animals ​+ ​OR MH animal studies OR TI animal model∗) …

### **Web of Science:**

(ALL=(((exercise) OR (Exercis∗ OR "physical activity" OR training OR fitness OR sport OR strength OR resistance OR running OR "race run∗" OR racerun∗ OR walk∗ OR cycle OR cycling OR bicycl∗ OR bike OR rowing OR aerobic OR anaerobic OR endurance OR swim∗ OR aquatic∗ OR dance OR dancing OR yoga OR "tai chi" OR Pilates OR "para sport" OR "active video gaming" OR exergam∗)))) AND ALL=((("cerebral palsy") OR ("Cerebral Palsy" OR athetoid OR ataxic OR athetosis OR dyskin∗ OR dyston∗ OR choreo-athetotic OR spastic OR hypertonia)))

Filters: 1980, clinical trial.

### **EMBASE:**

'exercise'/exp OR exercis∗:ti,ab OR 'physical activity':ti,ab OR training:ti,ab OR fitness:ti,ab OR sport:ti,ab OR strength:ti,ab OR resistance:ti,ab OR running:ti,ab OR 'race run∗':ti,ab OR racerun∗:ti,ab OR walk∗:ti,ab OR cycle:ti,ab OR cycling:ti,ab OR bicycl∗:ti,ab OR bike:ti,ab OR rowing:ti,ab OR aerobic:ti,ab OR anaerobic:ti,ab OR endurance:ti,ab OR swim∗:ti,ab OR aquatic∗:ti,ab OR dance:ti,ab OR dancing:ti,ab OR yoga:ti,ab OR 'tai chi':ti,ab OR pilates:ti,ab OR 'para sport':ti,ab OR 'active video gaming':ti,ab OR exergam∗:ti,ab.

AND.

'cerebral palsy'/exp OR 'cerebral palsy':ti,ab OR athetoid:ti,ab OR ataxic:ti,ab OR athetosis:ti,ab OR dyskin∗:ti,ab OR dyston∗:ti,ab OR 'choreo athetotic':ti,ab OR spastic:ti,ab OR hypertonia:ti,ab.

AND.

'clinical trial'/de OR 'randomized controlled trial'/de OR 'randomization'/de OR 'single blind procedure'/de OR 'double blind procedure'/de OR 'crossover procedure'/de OR 'placebo'/de OR 'prospective study'/de OR ('randomi?ed controlled' NEXT/1 trial∗) OR rct OR 'randomly allocated' OR 'allocated randomly' OR 'random allocation' OR (allocated NEAR/2 random) OR (single NEXT/1 blind∗) OR (double NEXT/1 blind∗) OR ((treble OR triple) NEAR/1 blind∗) OR placebo∗

### **PEDro:**

Exercise AND “cerebral palsy” AND “Clinical trial”
